# The bat meat chain and perceptions of the risk of contracting Ebola in the Mount Cameroon region

**DOI:** 10.1186/s12889-020-08460-8

**Published:** 2020-05-01

**Authors:** Efuet Simon Akem, Ngambouk Vitalis Pemunta

**Affiliations:** 1 Department of Sociology and Anthropology, University of Buea, Buea, Cameroon; 2 School of Public Health and Community Medicine, The Sahlgrenska Academy, University of Gothenburg, Box 453, 40530 Göteborg, Sweden

**Keywords:** Bat, Ebola, Gender, Hunting, Intersectionality

## Abstract

**Background:**

Local people’s interaction with bats render them vulnerable to Ebola Virus Disease (EVD). This paper examines perceptions of risk involved in the hunting, handling, processing and consumption of bat meat in the Mount Cameroon region of Southwest Cameroon. It focuses on the myriad cultural beliefs, gendered patterns of activity and institutional arrangements in which the bat meat production chain is embedded.

**Methods:**

We conducted 30 ethnographic interviews with a sample of purposively selected men and women involved in the bat meat trade. The interviews were tape recorded, transcribed verbatim and inductive analysis was performed on the data.

**Findings:**

The findings suggests that more urban men than villagers and hunters consume bat meat. Different practices and behaviours expose the mostly uneducated, young, single men and women to the risk of Ebola infection depending on their differential level of intervention in the human-bat interaction and value chain linking hunters, sellers and customers. The killing of bats with the mouth during hunting expose hunters (young men) while the preparation of bat carcasses for consumption also put women, (mostly young and unmarried) at risk.

**Conclusions:**

This study demonstrates that the complexity and nuances of gender, poverty and Ebola outcomes predispose some marginal groups to the risk of infection with zoonotic diseases. There is the need to improve public health intervention and health education among the rural masses in the Mount Cameroon region.

## Background

The hunting of bushmeat—the meat of wild animals—is a common and ancient practice in rural African villages, particularly in forested areas. Bushmeat has long been a delicacy among forest-dwelling peoples in Africa [1]. The African bushmeat trade is estimated annually to generate millions of American dollars [2]. In Western and Central Africa, the demand for bushmeat is estimated at 4.5 million tons per year and is rising. Sharply rising consumption of bushmeat is not a problem exclusive to Africa. For instance, demand is expected to quadruple in the Amazon basin [3]. is not only skyrocketing, it is projected to quadruple the demand for bushmeat in the Amazon while in the Congo Basin, the consumption of wildlife meat is estimated at 4.5 million tons a year [4].

The hunting and eating of bushmeat by humans however, carries a substantial risk for cross-species transmission of disease [3, 4]. Direct and indirect contact with bats differentially predispose various social actors to the risk of infection from zoonotic diseases. These diseases are a significant contributor to the emerging burden of disease outbreak and a growing threat to global public health. The interaction of human and wildlife has been considered as an important vector of wildlife infectious diseases that are transmissible to humans [3, 4]. Since the recent Ebola Virus Disease (EVD) outbreak 2014–2016, the resurgence of infectious diseases have received heightened attention [3]. Most (75%) of emerging infectious diseases are zoonotic. They are “mainly of viral origin and likely to be vector borne” [3]. Little attention has, however, been paid to gender roles and cultural practices that stand at the human-nature interface such as the hunting and consumption of possible animal vector carriers (in this case the bat).

Gender refers to socially defined and learned male and female behaviours that shape the opportunities that one is offered in life, the roles one may play and the kinds of relationships that one has. It is distinct from sex, which is a biologically determined and fixed set of characteristics for men and women. It is also distinct from- though closely linked with- sexuality, which is the “social construction of a biological drive” that is defined by how, why, and with whom one has sex [5]. Since gender is a social construct, the differences between men and women may vary from place to place. Springer and associates have conceptualized sex/gender as a domain of complex phenomena that are simultaneously biological and social, rather than a domain in which the social and biological “overlap” [6].

This paper draws on intersectionalist theory [7, 8] to investigate the gendered risk of contraction of EVD among bat hunters and sellers in the Mount Cameroon region (MCR) of Southwest Cameroon and in the wake of the 2014–2016 Ebola outbreak in West Africa. Although Cameroon has not yet been affected by the Ebola virus, it cannot be complacent because three of the country’s neighbours have recently experienced Ebola outbreaks. Gabon and the Democratic Republic of Congo experienced these outbreaks between 2001 and 2003 and a case was reported in Nigeria in 2015. Although the outbreaks in the recent past did not extend to Cameroon, the country was active in prevention efforts, which were restricted to sensitization, sealing of frontiers and restrictions of persons traveling from or to Ebola stricken countries. The country also volunteered to serve as testing ground for Ebola trial vaccines [9]. Are gender roles and other socioeconomic characteristics intertwined with the procurement and preparation of bat meat? How does bat hunting differentially expose people (men and women) to Ebola disease? Specifically, how does the practices of hunting and handling bat carcasses expose rural communities in the MCR to Ebola disease and how do these practices differ by gender? This study seeks to gain a detailed ethnographic understanding of bat–human–disease interactions, spillover and risks in the light of local cultural backgrounds in the MCR in order to understand how to effectively respond in the face of an Ebola outbreak.

### The ecological and social context of Mount Cameroon region

This ethnographic study aims at examining various gendered roles in the hunting and processing of bat meat in the MCR area where pathogens are entangled with human social worlds. Merrill Singer has emphasized a biosocial or biocultural approach to the understanding of infectious disease. In contrast to a strictly biomedical framework, her core argument is that infectious diseases cannot be understood through biology alone but must rather, be considered within the context of the cultural and social worlds humans inhabit [10].

Mount Cameroon, which partly constitutes the Mount Cameroon National Park (MCNP), is one of Africa’s largest mountains and volcanoes. It rises to 40,100 m above sea level [11]. The MCNP covering 58.178 ha and located between 4.055° - 4.378° N and 9.031° - 9.294° E was created in 2009 with the goal of promoting alternative sources of livelihood to the local population. It lies between 3°57′- 4°27′ N and 8°58′-9°24′E on the coast, and in the Gulf of Guinea. It is a huge volcanic mass with its long axis (about 45 km long and 30 km wide) running SW to NE. It has a high proportion of tropical forest, a result of high annual rainfall ranging from 2000 to 10,000 mm/year at the coast [11, 12]. It is densely populated with an estimated 450,000 inhabitants, living in urban and semi-urban areas, but mostly in the 41 villages bordering the MCNP [11].

An estimated 75% of the population are dependent on the exploitation of land and forest resources for their livelihood. The indigenous Bakweri and Bomboko make up a meagre 23% of the population [11, 12]. In some areas, e.g. in the north of the MCNP, migrants represent more than 90% of the population [12].They control most of the farmland, especially within the MCNP and the remnant Bomboko Reserve [13]. Most bat hunters are immigrant young boys—mostly of Bafwum origin from the Northwest Region of Cameroon. The Mount Cameroon area and its environs is characterized by high population density, rich volcanic soils and abundant precipitation [10]. In the Mount Cameroon forest zone, agriculture is the most important economic activity for both the indigenous people and local and immigrant groups.

The high rate of hunting/or poaching in the Mount Cameroon Forest Region has led to frequent human-wildlife contact. Fifty three (53) per cent of global Emerging Infectious Disease (EID) outbreaks from 1996 to 2009 were in Africa, yet the continent lags behind severely in infectious disease detection and emerging epidemic warnings [14, 15]. According to the Interagency Coalition on AIDS and Development for every HIV-positive young man (15–24 years), there are three HIV-positive young women [15]. Young women are disproportionately affected by the HIV/AIDS pandemic because of their inability because of differences in power between genders to negotiate safe sexual practices and because of their biological vulnerability [16, 17]. Knowledge of cultural practices related to the transmission of Ebola from its primary reservoir is deeply understudied, poorly understood and patchy but crucial in health promotion initiatives [4].

Human-wildlife (including bat) contact increases the risk of zoonotic pathogens in the MCR. Colonies of straw-colored (*Eidolon helvum*) and *Epomophorus gambianus* fruit bats have been identified as living in close proximity with humans [18–21] providing ecosystem services for residents but also transmitting diseases to humans (“ecosystems disservice”) [22]. Colonies of bats live in fruit trees including among others mango (*Mangifera indica*), guava (*Psidium guajava*) and pawpaw (*Carica papaya*) [22] around homesteads, in farms and in the forest. During the fieldwork for this study we also observed local hunting and consumption of the abovementioned bat species which are suspected to be reservoirs of several zoonotic or potentially zoonotic infections of significant public health significance [21–24] such as henipavirus, lyssavirus and EBV [23]. Additionally, the people’s main activity of subsistent and largescale agriculture expose them to scratching, bites, urine and droppings of bats. A combination of changes in human behaviour (underpinned by increasing human populations) and the spatial expansion of plantation agriculture by the Cameroon Development Corporation (CDC), Del Monte Inc. and private individuals, direct contact with bats through hunting, selling, and/or eating might provide great opportunity for zoonotic transmissions [19] This is the case of Cameroon’s Southwest Region [14] in general and the MCR in particular where there is the growing of palm oil, banana, rubber, tea, cocoa and subsistent food crops of up to 50 ha in size [11]. Fruit trees in farms and mountain caves provide roosts for significant colonies of fruit bats and avenues for human-bat contact that seem, as reported by health workers, to partly account for the high numbers of patients suffering from fevers and rabies in the area. Research findings from Ghana suggests that residents whose activities expose them to bats were more likely to experience fevers than their counterparts who do not frequently interact with bats [23]. Compounding the situation is the fact that in most remote areas of Cameroon including the MCR, the population lack basic infrastructures like roads, electricity and pipe borne water thereby forcing them to fetch water from streams or harvest water from rainfall that might be contaminated by bat urine and faeces.

The daily aerial movement of bats between feeding and roosting sites (between 5:30–6 pm and 4:30–500 am) means there is heightened risk of contaminating water harvesting systems through aerolisation of their droppings [23].The lack of these amenities have further deprived local people of ways to conserve meat and fish. Bush meat is the main source of protein in the area. Human-wildlife (including bat) contact is common in the area, thereby increasing the risk of zoonotic pathogens. The area also experiences weak public health facilities and health governance issues. Public health surveillance is poor and misdiagnosis or under-ascertainment of zoonotic diseases is likely since local people often downplay their risk of exposure to wildlife. This implies the likelihood of the lack of systematic surveillance [23–25] or effective diagnostic methods (misdiagnosis) of zoonotic diseases since local people often downplay the risk of their exposure to bats [23–25]. Misdiagnosis cannot be discounted due to lack state-of-the-art equipment for diagnosing zoonoses [22].

Local people’s interaction with, as well as the handling and consumption of bat meat renders them vulnerable to EVD. Following the 2014–2016 Ebola outbreak, the government of Cameroon banned the eating of bushmeat [26], including bats. Since then, bat hunting has become a secret undertaking across the country. In a typical patriarchal society where there is strict adherence to gender roles in the domestic, production and community settings [27], possible Ebola transmission routes are more likely to be sex and age-specific. Understanding the gendered and cultural dimensions of the spread of Ebola from its primary reservoir is vital in both ecological and public health contexts in order to identify possible pathways and prevention measures to any apparent threats.

### Theoretical framework

This study of how multiple identities related to the value chain–hunting, preparation and consumption—of bat meat treated social categories (gender, age, sex) as independent variables differently predispose men and women to the risk of infection with EVD and thereby generating main effects and interactions [8, 28], recognizes multiple identities as well as the influence of human social worlds in the analysis of the risk of disease contraction [26]. The focus of this “categorical approach” [7] to intersectionality deals with the complexity of relationships among multiple social groups within and across analytical categories…..The subject is multigroup and the method is systematically comparative ([7], P. 1786). Intersectionality [6, 8, 28] and ethno-ecological theories [29, 30] guided the collection of data for this study. The data analysis and interpretation were however, largely informed by intersectionalist theory.

Intersectionality is relevant in three ways for foregrounding the complexity of public health problems such as EBV and explaining disparate outcomes between social groups. First, it takes cognizance of the multiple interdependence, mutually constitutive and multidimensionality of social identities (group heterogeneity). Secondly, the focal or starting points are people from “multiple historically oppressed and marginalized groups” such as bat hunters and retailers (young men and women). Thirdly, the intersections of race, gender, and socioeconomic status—multiple social identities at the micro-level—crisscross with macro-level structural factors—poverty, racism, and sexism to result in different health outcomes [31]. Intersectionality embraces, rather than avoids, “the complexities that are essential to understanding social inequities, which in turn manifest in health inequities” [8, 28, 31]. An acknowledgement of “the existence of multiple intersecting identities is an initial step in understanding the complexities of health disparities for populations from multiple historically oppressed groups” [31].

In relation to the focus of this study, it can be observed that the risk of infection with EVD is intertwined with social and economic pressures of inequality. “These macro socio-structural dynamics intersect at the micro-level of individual experience to contour social attitudes and behaviours that place” [7] women and youths at risk of EVD infection due to their coming in contact with the carcasses of bats. This resonates with Bowleg’s view that multiple intersecting “identities at the micro-level reflects multiple and interlocking structural-level inequality at the macro-levels of society” [31].

Intersectionality theory provides a useful theoretical lens for examining the interactions between social identities and psychological characteristics of individuals. These includes—gender, socioeconomic status, age, perceptions, attitudes and behaviours as well as their health-related outcomes. It provides an analytical lens for exploring relationship between broader macro-level and individual level determinants and economically challenged and marginalized individual risk for infection [7] due to working arrangements based on gendered roles.

## Methods

### Data collection techniques

This study adopted a qualitative study design. We implemented ethnographic fieldwork using semi-structured interviews with key informants, participant observation and informal discussion sessions from May to October 2017 in the rural communities of Ekata, Bafia, Munyenge and Ekona at the foot of Mount Cameroon and Mbonjo village of the West Coast of the area. Through 2 hunters and 3 mobile vendors of bat meat we were acquainted with, we started meeting other participants and soliciting their voluntary participation in the study. Drawing on this network of participants (friends) we also employed purposive sampling [26]. According to Bernard, in purposive sampling, the researchers project the roles that certain respondents are likely to serve in their study. In the same vein, Burns ([32], P.465) notes that purposive sampling is useful if it serves the real purpose and objectives of the researcher[s] by enabling [them] to discover, gain insight and understanding into a particular phenomenon. To a limited extent, the selection of participants was informed by the “sampling logic” or “those that are representative of the total population of similar cases” ([33], P. 47).

The researchers followed, observed, and recorded the sequences of supply chain processes intertwined with the hunting and, transportation of bat carcases to the village, the market and the preparation of bat meat for consumption. The butchering, smoking, cooking and sharing of bat meat activities/practices were observed and recorded along gender lines. Interviews offer an opportunity for exploring a phenomenon in-depth. Furthermore, informants had the opportunity to state their opinions, motivations, and provide stories/narratives including accounts and explanations [33]. Each interview session lasted for approximately an hour. The interviews were conducted in either English or pidjin English. With the respondent’s permission, they were tape-recorded and transcribed at most 24 h thereafter. We also took copious notes. Respondents had the right to withdraw from the study at any given point in time. In fact, their rights to self-determination, confidentiality, and anonymity was ensured all through the research process.We obtained ethical clearance from the Ethics Committee of the University of Buea (Cameroon). Both written and oral informed consent was further obtained from each participant. Participant’s identities have been anonymized in the findings.

Ethno-ecological theory [29, 30] guided the collection of data but the analysis and interpretations were largely informed by the intersectionality theory of gender. Data analysis consisted of grouping the themes together in light of the objectives of the study (thematic analysis). In line with this method, we identified topics, ideas, or patterns from the data. We uncovered issues, problems, similarities and differences of opinion in the data. The following themes emerged— hunting and transportation of bat meat carcasses, preparation of bat meat, consumption of bat meat and local beliefs about bats. These themes provided in-depth understanding of the phenomenon under investigation [34].

## Results

### Hunters’ profile, methods and activities

During fieldwork for this study, thirty (30) participants were interviewed. They included 20 male bat hunters aged 22–33. They originated mostly from Mbonjo, Bafia and Ekona villages. Out of the 20 hunters, 19 of them had just a First School Leaving Certificate (primary school leavers) while one (1) was a graduate from the Department of Geography, University of Buea. They were all unemployed, single, smoked cigarettes and earning a livelihood from bat hunting. They go for hunting daily– especially at night from 11 pm to 4 am, except on days on which heavy rainfalls or on very windy days. They stated that heavy rainfall prevents bats from flying around freely. Furthermore, 6 women involved in the preparation and selling of bat meat were interviewed. The women aged 20–28 were singles. Two (2) of them openly declared they are into casual sexual activities with some of the hunters. Four (4) individual consumers were interviewed. The different responses of hunters and sellers showed that they hunt/sell bat meat for food and money.

The method for catching bat in the Mount Cameroon area is mainly by the use of nets. Nets are collected from fishermen in Limbe- a seaport town. These are usually over used and waste fishing nets that the fisher men consider not strong enough for fishing. Bat hunters get these nets sometimes free or offer a generous, unspecified token. Besides the nets, the hunters use cutlasses, touch lights, bags, 4 wooden poles and several other hard sticks.

Although an individual can carry the fresh kills, the hunters consider this as very stressful. Ideal in the study area is a hunting ‘team’ comprised of two persons. They use the African proverbs “when people are together, they hunt tiger together”. The hunters pair themselves up and identify a suitable hunting territory in the bush or forest. The choice of the hunting site depends on the characteristics of the forest or area, which include two tall trees that are usually about 10 m apart, sparsely or densely packed with assorted varieties of bush plants. In between these two trees, they construct a wooden elevation of about 2 m high in the shape of a barn (locally called `bander`). These four fax-sticks barns are planted on the ground, criss-cuts with several small but very strong sticks. This serves as an elevator which one hunter climbs on it to maintain a certain height. A spread net sheet of about 4 m length and 3 m width is hung on two Indian bamboo sticks. Indian bamboos are often chosen because of their lightweight and are strong.

Beneath the barn, another hunter mimics (cries/imitates the sound) of a bat. The passing bat is lured by this sound and follows the direction, hoping the crying bat has found food. As they fly towards that direction, they enter into the net. This first set is caught but not be killed. To lure the other bats, one of the hunters use the bats that were caught earlier. This is done by biting and pressing the neck, and as the bat screeches or screams, the sound further invites other bats. This exercise is done repeatedly. Bats that are selected for this purpose are usually big in size and are locally called `*capitol`* (meaning captain). When these bats become weak and their voices are not loud, they are exchanged for another `*capitol`* and the process continues. The hunters complain that bats are very strong, dangerous and very difficult to handle when alive. Once the bat is removed from the nets, the hunters wrench the vertebral column (around the neck) of the bat with their teeth while pressing the head with lot of force for it to die. Amazingly, the hunters do this with each of the bats caught.

### Intersectionality and the transportation of carcasses

In the bush, the bats are put into a bag. They are transported to the kitchen on the head or the back. However, hunters from distance places like the West coast village of Mbonjo transport their catch in a taxi to the nearest town- Limbe where it is delivered to an agent. The agents take it into another town, Muyuka. Thereafter, the agents dispatch the bats to Bafia, Munyenge and Ekata villages for preparation and consumption. Upon the arrival of the villagers in the village (as early as 5:30 am, they all gather on a particular spot, count and tie their catch into bundles of 3–4 per bundle depending on the size. They sometimes put together 8 very small ones per bundle. A bundle sells at 1000Fcfa^1^ (approx. $10). Hunters in the West coast villages assign one person to take all the bats caught for the day to the buyers in the predominant bat eating villages of Bafia, Munyenge, Ekata and Ekona.

### Intersectionality and preparation of bat meat

The preparation of bat carcasses for consumption takes several intertwined stages. For instance, fixing, burning/ roasting, butchering/slicing, washing, steaming, frying, and preparing bat sauce ingredients. Unemployed and unmarried women predominantly carry out these processes. It is largely a complimentary mode of survival. When the fresh bats from the bush get to the women, they organize the kitchen by creating space for work to begin. Friends, sisters and children voluntarily assist in the series of preparatory activities that ensue. Fixing comprises of handling and removing the wings of the bat. The villagers use two methods, with the mouth and knife. According to the hunters, using the mouth is the traditional and most appropriate way of doing it. To them it is better to disentangle the bones of the wings with the teeth because it is very strong.

After disentangling the wings, fire is set with considerable flame and a wire gauge is placed on it. Between 8 and 10 bat carcasses are laid on the gauge. The bats cook for 5–7 min during which the carcasses are turned repeatedly on the fire until the hair is burnt. After slicing open the stomachs, the bat carcasses are left intact. After operating the stomach, the bat carcasses are washed with warm water to render them very clean. In order to effectively clean them, warm water is compulsory for washing bat carcasses. The second washing is done but with fresh water. Thereafter, the bat meat is boiled for about 20 min with salt. To prepare the sauce, a combination of palm oil and groundnut oil are placed on the fire for about 5 min. Later, other condiments like tomatoes, cube, onion, pepper, ginger and garlic are mixed and stirred. In about 10 min, the bat meat is then poured on the tomato sauce with a small quantity of water and allowed on the fire for about 5 min.

### Intersectionality and bat meat consumption

The hunters sell most of the bat meat. Bat meat is mostly consumed in bars rather than at home. Men and women, old and young, consume bat meat. Most consumers (adult men and sometimes women) are people who frequent drinking spots. They eat bat meat while drinking beer, but sometimes some individuals buy and take home. This finding resonates with a recent study that suggests differences between age and sex in the frequency of bushmeat consumption in six West African cities from four countries. Overall, they found similar patterns in all cities; 62.2% males and 72.1% females stated that they would not consume bushmeat at all, though 12.8% males and 8.8% females liked bushmeat and regularly consumed it [2].

Women move round the village hawking prepared bat meat. Their main targets are beer parlors and traditional palm wine bars. Consumption is very high in the months of March, April and May. This is the cash crop season when villagers have money after selling cash crops (cocoa, coffee and palm oil). Bat meat is sometimes consumed with corn fufu, plantain, cocoyam etc. at home. Generally, female mobile street vendors in the village sell cooked bat meat.

### Local beliefs about bat and EVD

Most non-consumers of bat meat consider it as an animal that represents people’s totem. Some claim that a bat looks wild and frightful. In a light-hearted manner, others stated that the name bat sounds `bad`, and question why people should eat something that is ‘bad’. A study from Ghana suggests the existence of a cultural prohibition against pregnant women consuming bats because bats are believed to have no rectum and vomit their excreta, they are therefore keen to minimize the risk of transmitting the “vomiting habit” to their children after birth [22].

As for the causes and origin of the virus, majority of the informants think the virus exists but that Western governments fabricated it. Very few respondents believe that Ebola is a natural disease. Some of the informants hold that the cause of the EVD is witchcraft and that it is some sort of poison from people with malevolent intentions. Those who continue to eat bat meat believe that the virus exits but elsewhere, not in their forest. Meanwhile, those who think the EVD originated from the West hold that the aim is to discourage them from killing animals. This has resonances with the belief in the Ve Golokuati Township in the Volta Region of Ghana who concede that bats do not harbour disease. Their view is informed by the non-occurrence of any strange disease outbreaks in the town since the coming of bats [22]. Below follow some illustrative interview excerpts:

*These diseases are foreign. They are occurring somewhere else and not here in our forest but we really need to be careful (keep guard) (Farmer and bat meat consumer).*

A hunter expressed his frustration thus:

The *coming of Ebola and other wildlife diseases has touched the very core of our economic, cultural and nutritive life*.

*We don’t know the actual situation here, if they (Governments, NGOs etc) do not say anything, we do not know anything*. (A female bat meat consumer).

Most bat hunters and the larger community expressed similar views. To the local people, the EVD is an illusion. Generally, the informants were ignorant about the causes and origin of Ebola. Whereas most women lacked this knowledge because the area lacks electricity (thus limited access to radio and television news), men always gather in bars and get information from people coming from the cities while women are generally isolated in the kitchen. They do not have such opportunities for group life, conviviality and access to information.

## Discussion of findings

How does the procurement, preparation and consumption of bat meat differently expose men and women in rural communities in the MCR to EVD?

The findings of the study showed that most bat hunters and sellers are single, have little education and are otherwise jobless. This is in consonant with findings from Ghana where men, often with little education are said to dominate bat hunting, selling, preparation or its consumption [35, 36]. The actors in the bat meat chain suffer from multiple vulnerabilities that predispose them to the risk of contracting zoonotic diseases [7, 8, 26].Their young ages (22–33 males and 20–28 females) contradicts the older ages of participants in the bushmeat trade, the inverse relationship and the dwindling youth interest in bat-bushmeat hunting observed in Ghana [23]. This may seem to suggest that formal education and employment will lead to the decline of bat bushmeat hunting and processing. This calls for the need to understand “the complexities of the socio-economic environments in which zoonotic outbreaks occur” [23] to appropriately ensure control and preventive measures [23].

The traditional technique of hunters using their teeth to kill the captured bat, the transportation of the killed bats on the back from the bush, the fixing of the wings using their teeth and the scratch they get from the bat put them at very high risk because of the contact with blood and body fluids through bites and scratches [22]. Furthermore, women involved in preparing the bat meat for consumption are inordinately exposed to blood and the contraction of zoonotic diseases, including EVD. The gendered division of labour (roles) in the bat meat trade between hunters (male) and vendors (female) is consistent with the cultural norms of Cameroonian and African societies [23].

Policymakers should be aware that low education and poverty has pushed these young men and women into the bushmeat trade. These, are, however, just a few among other disadvantageous consequences [36] that hunters and sellers of bat meat are predisposed to, and which exposes them to the risk of contracting zoonotic diseases including EVD. Public health interventions should emphasize how gender and poverty shape, intersect and predispose certain women and young unemployed men to the risk of contracting re-emerging zoonotic diseases from bats. This situation calls for the need for a more holistic vision of risk and its operationalization. The social and cultural practices (behaviours and experiences) of hunters, sellers and consumers of bat meat rather than identity categories differentially expose them to the risk of infection from EVD. In the fight against re-emerging zoonotic diseases, behaviour change based on, rather than social categorization should ideally be used to operationalize risk [7].

Men and women are involved in the myriad activities required to bring bat meat through different stages of production (hunting, preparation of carcasses for transportation, cooking—a form of value-added processing for delivery to the final consumers. This value chain requires different skills, market-based relationships among different social actors, which inordinately produce different risks and gains for different stakeholders [37]. We were partly interested in accounting for the gendered pattern of vulnerability from the hunting and consumption of bat meat in our data. In a society that adheres to strict gender roles in the domestic, production and community settings [27], possible Ebola transmission routes are more likely to be sex and age-specific.

Independent of gender, participants involved in the production chain of bat meat activities are exposed to bat bites, scratches, urine, secretions and blood, which exposes them to diseases transmitted by bats. However, being male and young in our study area creates additional vulnerability because of the different gendered roles men play in the bat hunting, transportation and sometimes fixing process. Hunting is considered a masculine activity that involves physical and spiritual powers [38, 39]. The process of killing and fixing bat carcasses with the month/teeth poses a potential danger of contracting a zoonotic disease because of the high probability of having an open skin and possible blood transmission. Each of these settings could also provide entry points for the Ebola virus. There is also a high probability of female vulnerability from the virus in the rural, agricultural areas of Mount Cameroon. The preparatory and cooking roles of women and children, especially the girl child can expose them to sources of contamination within the household. The predominance of women as rural petty hawkers of bat meat further increases their vulnerability to the Ebola virus. Women and girls with unstable socio-economic circumstances face the challenge of simultaneously caring for small babies while working. For instance, mobile bat meat hawkers often strap babies on their backs, amidst limited access to hygiene facilities.

Prior to the recent Ebola outbreak, the consumption of bat meat was common in big towns like Limbe, Muyuka and Tiko. This drastically reduced because the outbreak of the Ebola virus in West Africa that subsequently led to the ban on bat hunting and consumption in Cameroon. On the contrary, the hunting and trading of fried bat meat was widely observed in remote rural areas including Bafia, Munyenge and Ekata. This seems to suggest that unlike rural people (mostly poor), urbanites (rich compared to rural dwellers) may be more willing to adjust and cope with perceived threats of disease infection.

The supply chain of bat meat is heavily influenced by climate change and the recent Ebola crisis of 2014–2016. According to local ethno-ecological knowledge, too much rain is not conducive to the hunting of bats. During the wet season, especially in the months of July and August, bat hunting is rare because of the heavy rains. During such periods, the bats fly very high in the sky, targeting fruits from tall trees in the forest. Whereas, fruits is less abundant in the dry season, which motivates bats to then fly low and even near dwelling settlements. Because of the climate change in the MCR due to climate vulnerability and change, bat hunters migrate to different communities as the bat themselves migrate in search of food and niche (ecological niche) [39]. The Ebola crisis and the ban on game meat led to the avoidance of bat meat while suppliers (hunters) were afraid of criminal sanctions from the government [9].

Local people claim bats are agents of witches. As a result, bats were considered as protecting their harvest. In the same vein, the local people considered night hunting as dangerous because they believe witches and wizards are active at night. Generally, however, the local people are losing their traditional hunting lifestyle. Hunting is now done with almost complete disregard to the age-old socio-cultural norms of the people. Some cultural traditions, which are no longer respected, used to help reduce pressure on bat species.

We were keen to know what the people do when they find wildlife carcasses or partly eaten fruits in the forest that were not killed or harvested by the individual/hunter. Local cultural norms used to prohibit the eating of carcasses. Such animals were believed to cause ill luck and disease. This norm is no longer in vogue today. This change can be explained by culture change, economic hardship, bushmeat scarcity and therefore, the increased monetary value of bushmeat including bats in the area. Most participants admitted they have seen dead animals in the forest, which they collected and consumed. Those who take home the animal carcasses argue that most of the animal carcasses found in the forest are because:

- An ill targeted bullet from a hunter, which does not permit it to die at the spot. So the animal can escape to a different part of the forest and later die;
- The animal happened to escape from a snare trap. After a long fight, the animal cuts the snares but later on suffers from internal bleeding and dies;
- Some wild and stronger carnivorous animal can possibly kill a small or less powerful animal in the forest. Like many of our ethnographic partners, a hunter gave us to understand that tiger, gorilla, chimpanzee, and lion are killing most of the smaller animals in the forest. To him, most of these dead animals are rodents;
- Poisonous animals such as snakes and other reptiles can also bite and kill an animal.

In recent times, animals are also being caught using poisoned baits. This issue has raised considerable concern. It has however, been recently suggested that chemical poisons [30] contaminate most bushmeat. Despite this suggestion, people who collect the carcasses of dead animals for consumption are unable to determine the exact cause of death. All the reasons they forward as the causes of the death of these animals are guesswork. Thus, taking back such animals can be of potential danger to the individual and community health, should in case the animal is contaminated by infectious viruses. Some of these animal carcasses, which are in most cases almost in a state of decomposition, are still consumed. They believe that animals that are partly decomposed taste different and nice. That is, semi-decomposed meat has a special taste that they cherish. The carcasses of dead animals are mostly prepared with beans and porridge food.

## Conclusion

Hunters no longer respect traditional norms that ensured safeguards against wildlife diseases. This is reflected in the dwindling of taboos and norms related to the hunting and consumption of bat meat. Most bat hunters are single, have little education and jobless. It can be stated that they suffer from multiple vulnerabilities that predispose them to the risk of infection from human-animal contact [7, 8]. The traditional technique of using their teeth to kill the captured bat, the transportation of the killed bats on the back from the bush, the use of their teeth to fix the wings and the scratch they get from the bat, put them at very high risk because of the contact with blood. Beyond the hunting and transportation of bat, in tandem with gender roles, young women are involved in the preparation processes (fixing, burning, slicing and cooking). These activities expose them to the risk of the several contact points with blood from bat meat. Gender plays a role in susceptibility to attack by Ebola. This is the case because this society adheres to strict gender roles in the domestic, production and community settings thus, making Ebola transmission routes more likely to be sex and age-specific. It can therefore, be stated that women are embedded in social, cultural, and historical contexts and institutional practices [40] that expose them to the risk of infection with zoonotic diseases.

As bushmeat, bat meat has largely disappeared from sales in main towns like Buea and Limbe for fear of the risks of zoonotic pathogens associated with bush meat, including bat. Bat meat is however, still being consumed in remote villages.

In line with intersectional theory, most youths and women who are exposed to bats need resources to effectively address the structural and material inequities affecting their life opportunities and predisposing them to infection from bat-human contact. It is essential for policymakers to recognize that poverty, low education that pushes these young men and women are just a few among other disadvantageous consequences [36].

There is the need in public health interventions to focus on how statuses including gender and poverty shape and predispose certain women and young unemployed men to the risk of contracting zoonotic diseases from bats. Oppression (risk) operates through a series of interlocking systems that crisscross intersecting conventional identity categories [5–7]. In line with Dworkin, the women-at-risk paradigm overlook men’s relative positioning vis-à-vis patriarchy [41]. Behaviour and experiences rather than identity categories expose these individuals to the risk of infection from EVD. Risk should ideally be operationalized in terms of behaviour rather than through social categorization [7]. This is not to undermine other categories because there is also the role of powered disease “transmission and infection”… that are intertwined with social and economic relations of inequality [41].

In the study area, bat-human contact and the risk of contamination occur in myriad ways. Apart from farming, hunting and processing of bat bushmeat, the harvesting of rainwater after long spells of the absence of rain that may be contaminated by bats droppings on rooftops. Largescale agriculture has led to habitat encroachment, sometimes forcing bats to utilize human structures as roosts thereby exposing humans to odour from faecal droppings, urine, aerolisation of saliva, and glandular body secretions [22].

## Supplementary information

**Additional file 1.** Interview Guide. This document contains the questions used in the interviews with participants of the study.

## Data Availability

In tandem with standard research practice and as promised respondents, the tape recorded interviews were destroyed after transcription. To further protect the anonymity of participants, the datasets generated and analyzed during the current study are not publicly available. Redacted versions are, however, available from the corresponding author upon reasonable request.

## Abbreviations

AIDS: Acquired Immune Deficiency Syndrome
CDC: Cameroon Development Corporation
EVD: Ebola Virus Disease
EID: Emerging Infectious Disease
HIV: Human Immuno-deficiency Virus
IUCN: International Union for the Conservation of Nature
MCNP: Mount Cameroon National Park
MCR: Mount Cameroon Region
NTFPs: Non-timber Forest Products
WHO: World Health Organisation

## Footnotes

1 Central African Francs
