# Supplementary material for: The bat meat chain and perceptions of the risk of contracting Ebola in the Mount Cameroon region
Source: BMC Public Health. 2020 May 1;20:593. doi: 10.1186/s12889-020-08460-8 (PMC7193336; doi:10.1186/s12889-020-08460-8)
Supplement: Supplementary file 1 — Additional file 1. Interview Guide. This document contains the questions used in the interviews with participants of the study. [file 12889_2020_8460_MOESM1_ESM.docx]

**Interview guide – Bat hunters (participants)**

Can you start with telling me about yourself?

- Age, tribe/region of origin?

- What is your reason for hunting bats?

- How long have you been hunting bats?

---How is the hunting of bats organized? How are bats hunted?

---Where does the hunting take place?

----Do you hunt other rodents, which are they?

---Can you describe the preparation and hunting process?

---How is the hunted bat meat transported to consumers?

----Who are your customers?

----How is bat meat prepared?

----Who does the preparation?

---Do you think the hunting of bat meat exposes you to the ebola virus disease? How?

---Who is most exposed to the risk of contracting diseases by coming into contact with bat meat?

---- Why are you still hunting and selling bat meat despite the government ban?

---Why is it more appropriate to use your teeth in breaking the back/bones of a bat?

----What are the risk of hunting bat meat?

---How do people perceive those who eat bat meat?

---Apart from hunting bats, which other activities are you involved in?

**Vendors**

---How did you come about procuring and selling bat meat?

---Who supplies you the bat meat that you prepare and sell?

----What are the risks in the preparation of bat meat?

----Is the preparation and consumption of bat meat linked to the present ebola outbreak?

--If yes, how?

-----What is your reaction to the government ban on the consumption of bat meat?

-
